# Supplementary material for: Genomic evidence for global ocean plankton biogeography shaped by large-scale current systems
Source: eLife. 2022 Aug 3;11:e78129. doi: 10.7554/eLife.78129 (PMC9348854; doi:10.7554/eLife.78129)
Supplement: Transparent reporting form [file elife-78129-transrepform1.pdf]

Westbrook Centre
Milton Road
Cambridge CB4 1YG, UK
P 01223 855340
W elifesciences.org
T @elife

eLife's transparent reporting form

We encourage authors to provide detailed information *within their submission* to facilitate the interpretation and replication of experiments. Authors can upload supporting documentation to indicate the use of appropriate reporting guidelines for health-related research (see EQUATOR Network), life science research (see the BioSharing Information Resource), or the ARRIVE guidelines for reporting work involving animal research. Where applicable, authors should refer to any relevant reporting standards documents in this form.

If you have any questions, please consult our Journal Policies and/or contact us: editorial@elifesciences.org.

Sample-size estimation

- You should state whether an appropriate sample size was computed when the study was being designed
- You should state the statistical method of sample size computation and any required assumptions
- If no explicit power analysis was used, you should describe how you decided what sample (replicate) size (number) to use

Please outline where this information can be found within the submission (e.g., sections or figure legends), or explain why this information doesn't apply to your submission:

> The *Tara* Oceans expedition was designed to optimize sampling at a global scale over a three-year period, operating within the limitations of sampling logistics, boat travel, ocean physical characteristics and national and international laws. Full details on the sampling design and protocols are presented in Pesant *et al*. (2015) https://doi.org/10.1038/sdata.2015.23

Replicates

- You should report how often each experiment was performed
- You should include a definition of biological versus technical replication
- The data obtained should be provided and sufficient information should be provided to indicate the number of independent biological and/or technical replicates
- If you encountered any outliers, you should describe how these were handled
- Criteria for exclusion/inclusion of data should be clearly stated
- High-throughput sequence data should be uploaded before submission, with a private link for reviewers provided (these are available from both GEO and ArrayExpress)

Please outline where this information can be found within the submission (e.g., sections or figure legends), or explain why this information doesn't apply to your submission:

> The *Tara* Oceans expedition was conducted once; biological replicates are not available. Throughout our manuscript, we confirm principal results on three different data sets extracted from the same set of samples: metagenomes, metabarcodes and imaging data. These analyses are presented in the figures and figure supplements. For certain analyses, some samples with different oceanographic properties were considered separately from others; these criteria are described in the methods sections corresponding to each analysis (Environmental differences among genomic provinces; Correlations of β-diversity, T_{min} and environmental parameters; Community turnover in the North Atlantic).

Statistical reporting

- Statistical analysis methods should be described and justified
- Raw data should be presented in figures whenever informative to do so (typically when N per group is less than 10)
- For each experiment, you should identify the statistical tests used, exact values of N, definitions of center, methods of multiple test correction, and dispersion and precision measures (e.g., mean, median, SD, SEM, confidence intervals; and, for the major substantive results, a measure of effect size (e.g., Pearson's r, Cohen's d)
- Report exact p-values wherever possible alongside the summary statistics and 95% confidence intervals. These should be reported for all key questions and not only when the p-value is less than 0.05.

Please outline where this information can be found within the submission (e.g., sections or figure legends), or explain why this information doesn't apply to your submission:

> Statistical tests applied are described in the main text, the methods and the legends of figures and figure supplements associated with each analysis. The counts of samples analyzed are represented visually on geographic maps (as in Figure 1 and Figure 1–figure supplement 1b) and summarized in Figure 1–figure supplement 3a.

(For large datasets, or papers with a very large number of statistical tests, you may upload a single table file with tests, Ns, etc., with reference to sections in the manuscript.)

Group allocation

- Indicate how samples were allocated into experimental groups (in the case of clinical studies, please specify allocation to treatment method); if randomization was used, please also state if restricted randomization was applied
- Indicate if masking was used during group allocation, data collection and/or data analysis

Please outline where this information can be found within the submission (e.g., sections or figure legends), or explain why this information doesn't apply to your submission:

> We did not perform comparisons of samples allocated into experimental groups.

Additional data files ("source data")

- We encourage you to upload relevant additional data files, such as numerical data that are represented as a graph in a figure, or as a summary table
- Where provided, these should be in the most useful format, and they can be uploaded as "Source data" files linked to a main figure or table
- Include model definition files including the full list of parameters used
- Include code used for data analysis (e.g., R, MatLab)
- Avoid stating that data files are "available upon request"

Please indicate the figures or tables for which source data files have been provided:

eLife Sciences Publications, Ltd is a limited liability non-profit non-stock corporation incorporated in the State of Delaware, USA, with company number 5030732, and is registered in the UK with company number FC030576 and branch number BR015634 at the address Westbrook Centre, Milton Road, Cambridge CB4 1YG.

Metagenomic and metabarcoding sequencing reads have been deposited at the European Nucleotide Archive under accession numbers provided in Supplementary Table 1. Contextual metadata of *Tara* Oceans stations are available in Supplementary Table 2. Metagenomic dissimilarity, OTU community dissimilarity, imaging community dissimilarity, simulated travel times, geographic distances and MAG dissimilarity are provided in Supplementary Tables 3-18. All Supplementary Tables, in addition to Datasets 1-4 (tables of 18S V9 barcodes and OTUs, the V9 reference database and MAG abundances) are available on FigShare at the following URL: https://doi.org/10.6084/m9.figshare.11303177. Images and their taxonomic assignations are accessible within Ecotaxa (https://ecotaxa.obs-vlfr.fr/prj/377).

eLife Sciences Publications, Ltd is a limited liability non-profit non-stock corporation incorporated in the State of Delaware, USA, with company number 5030732, and is registered in the UK with company number FC030576 and branch number BR015634 at the address Westbrook Centre, Milton Road, Cambridge CB4 1YG.
